# Systematic review and critical methodological appraisal of community-based falls prevention economic models

**DOI:** 10.1186/s12962-022-00367-y

**Published:** 2022-07-16

**Authors:** Joseph Kwon, Hazel Squires, Matthew Franklin, Tracey Young

**Affiliations:** School of Health and Related Research, University of Sheffield, Regent Court (ScHARR), 30 Regent Street, Sheffield, S1 4DA England UK

**Keywords:** Geriatric public health, Falls prevention, Decision modelling, Economic evaluation

## Abstract

**Background:**

Falls impose significant health and economic burdens on community-dwelling older persons. Decision modelling can inform commissioning of alternative falls prevention strategies. Several methodological challenges arise when modelling public health interventions including community-based falls prevention. This study aims to conduct a systematic review (SR) to: systematically identify community-based falls prevention economic models; synthesise and critically appraise how the models handled key methodological challenges associated with public health modelling; and suggest areas for further methodological research.

**Methods:**

The SR followed the 2021 PRISMA reporting guideline and covered the period 2003–2020 and 12 academic databases and grey literature. The extracted methodological features of included models were synthesised by their relevance to the following challenges: (1) capturing non-health outcomes and societal intervention costs; (2) considering heterogeneity and dynamic complexity; (3) considering theories of human behaviour and implementation; and (4) considering equity issues. The critical appraisal assessed the prevalence of each feature across models, then appraised the methods used to incorporate the feature. The methodological strengths and limitations stated by the modellers were used as indicators of desirable modelling practice and scope for improvement, respectively. The methods were also compared against those suggested in the broader empirical and methodological literature. Areas of further methodological research were suggested based on appraisal results.

**Results:**

46 models were identified. Comprehensive incorporation of non-health outcomes and societal intervention costs was infrequent. The assessments of heterogeneity and dynamic complexity were limited; subgroup delineation was confined primarily to demographics and binary disease/physical status. Few models incorporated heterogeneity in intervention implementation level, efficacy and cost. Few dynamic variables other than age and falls history were incorporated to characterise the trajectories of falls risk and general health/frailty. Intervention sustainability was frequently based on assumptions; few models estimated the economic/health returns from improved implementation. Seven models incorporated ethnicity- and severity-based subgroups but did not estimate the equity-efficiency trade-offs. Sixteen methodological research suggestions were made.

**Conclusion:**

Existing community-based falls prevention models contain methodological limitations spanning four challenge areas relevant for public health modelling. There is scope for further methodological research to inform the development of falls prevention and other public health models.

**Supplementary Information:**

The online version contains supplementary material available at 10.1186/s12962-022-00367-y.

## Background

The process of ageing encompasses multidimensional changes in physical and psychosocial domains [1]. Associated with ageing are risks of geriatric syndromes (e.g., falls, incontinence, delirium, and frailty) as symptoms of age-related impairments to multiple organ and physiological systems [2–4]. Around a third of people aged 65 and over (65+) fall each year [5]. As a geriatric syndrome, falls impose significant morbidity and mortality burdens [6], including fear of falling [7–9], depression [10], functional dependence [11–13], and fatality [14–16]. Falls can impose high costs on the health and social care systems [17–19], and on patients and wider society through out-of-pocket (OOP) care expenditure [20, 21], informal caregiver burden [22–24], and productivity loss [25, 26].

The National Institute for Health and Care Excellence (NICE) falls prevention clinical guideline (CG161) for England and Wales recommends that community-dwelling people aged 65+ are routinely screened for falls risk. High-risk individuals should be referred to multifactorial intervention involving multidisciplinary falls risk assessment, followed by tailored treatments including exercise, home assessment and modification (HAM), vision correction and medication change [5]. This proactive (i.e., initiated by professional referral) pathway may be supplemented by a reactive pathway for persons admitted to a medical facility for a fall (recommended by CG161), and self-referral to community programmes [27, 28]. Trial-based evidence suggests multifactorial and single-component community-based interventions significantly reduce the number of falls and/or fallers [29–31].

Commissioning of these community-based interventions should be informed by economic evaluations that consider the costs and consequences of any falls prevention strategy against the next best alternative use of resources [32]. Decision modelling is a vehicle for economic evaluation that can combine multiple epidemiological, intervention, and economic parameters from diverse sources [33]. Decision models have several advantages relative to economic evaluations alongside single clinical studies, such as the ability to incorporate long-term trajectories of falls risk, synthesise evidence from a range of sources, and evaluate all relevant intervention scenarios [34].

There are methodological challenges which are likelier to arise when economic evaluation is applied to *public health* rather than *clinical* interventions [35–38]. The former typically target general populations rather than narrowly defined patient groups, thereby generating significant heterogeneity in intervention access and outcomes across individuals and population subgroups; persistent variation over time generates dynamic complexity. Inequalities in outcomes across subgroups also become a normative issue and often an explicit policy objective to reduce. Moreover, public health interventions frequently require behavioural changes at the individual lifestyle and communal levels, raising issues in implementation. The determinants of health targeted by public health interventions are much broader than the biological mechanisms modified by clinical treatments, and include, for example, the physical/social environment. Public health interventions hence often require the involvement of non-healthcare stakeholders, and their evaluations should involve the tracking of wider non-health outcomes and non-healthcare costs. These challenges are particularly pertinent to decision modelling: first because models are well-positioned to incorporate key methodological solutions—e.g., expanding the evaluation horizon to account for dynamic complexity and appraising the efficiency-equity impacts of multiple intervention strategies; and second due to a concomitant risk that failing to address the challenges would produce less-than-credible model structures and results [39, 40].

Community-based falls prevention could be conceived as a representative public health intervention for several reasons. First, the multidimensional and syndromic nature of falls generates significant heterogeneity in falls risk among the general older population and the associated need for risk screening and multiple intervention pathways as noted. Second, falls are closely associated with frailty [41, 42], which in turn is closely associated with socioeconomic deprivation [43, 44]. Hence, the falls prevention strategy should consider social inequities of health across population subgroups. Third, falls prevention access is highly dependent upon older persons’ motives and professional behaviour [45–47] and therefore face significant implementation issues [48]. These features of falls prevention suggest that the aforementioned challenges to public health economic evaluation apply equally to falls prevention economic evaluations, particularly falls prevention economic models.

A systematic review approach can be used to critically appraise how existing falls prevention economic models have handled the methodological challenges, and thereby assist in the conceptualisation of future models [39]. A previous systematic overview of systematic reviews found that such critical appraisal was not performed comprehensively by the seven existing systematic reviews of community-based falls prevention economic evaluations [49]. For example, the extracted modelling features were limited to model type and brief summaries of data sources. Broader challenges for public health economic modelling were rarely considered, with only one review appraising how previous models incorporated wider costs and outcomes (e.g., productivity loss) [50], while another appraised how they considered issues of equity [51]. The reviews also covered heterogeneous sets of models such that their results do not constitute a comprehensive appraisal.

These findings motivated the current de novo systematic review, the methods and first part of results of which were recently published in a separate article [52]. The latter identified 46 models of community-based falls prevention, applied a methodological and reporting quality checklist designed by falls prevention experts [53], narratively synthesised methodological features relevant to falls epidemiology, falls prevention intervention and evaluation methods, and generated methodological and commissioning recommendations for falls prevention modellers and commissioners. The current article continues the narrative synthesis and appraisal, focusing on how the 46 models handled the challenges specific to public health economic modelling, and generates suggestions for further methodological research.

## Challenges to public health economic modelling

A previous systematic methodological review identified the following four key challenge categories to public health modelling [40]: (1) capturing non-health outcomes and societal intervention costs of falls prevention; (2) considering heterogeneity and dynamic complexity in health determinants and intervention need; (3) considering theories of human behaviour and implementation; and (4) considering issues of equity. These provide the analytical public health modelling challenges (PHMC) framework for appraising the falls prevention economic models identified by the systematic review and are described in more detail in this section. A caveat is that the challenges do not exhaust the range of methodological issues present within public health economic modelling but rather highlight the key ones discussed in the literature as identified by the systematic methodological review [40].

### Capturing non-health outcomes and societal intervention costs

Public health issues and interventions frequently affect non-health outcomes and expend resources outside the public healthcare system [36, 40, 53–55]. Prominent non-health effects of falls include: reduction in social wellbeing [56–58], often poorly captured in health status/utility measures [59]; OOP care expenditures (around 12% of annual care costs of fallers) [20]; loss in paid and unpaid productivity [26, 55] and the related loss in older persons’ wellbeing [60]; and informal caregiver cost (around 22% of annual care costs of fallers) [23] and health-related stress, particularly when the caregivers themselves are old/frail [61–63]. Meanwhile, falls prevention interventions incur societal costs, including: social stigma in participation (although this can also bring social benefits [64]), particularly in contexts where geriatric health promotion is uncommon [47]; private co-payments and costs (e.g., for transport); and time opportunity costs for participants and accompanying caregivers [65, 66]. Falls prevention may also bring benefits that chiefly accrue to the community rather than to individuals [67–69]; for example, community-wide participation can strengthen the community’s ability to organise other health promotion initiatives. The communal benefits should be weighed against the resources invested for social mobilisation, particularly those not reimbursed by the public sector (e.g., volunteer labour). Whilst the evaluation perspective will be dependent upon the decision-making context, it is likely useful to policymakers for models to capture as many of these outcomes and costs as possible.

### Considering heterogeneity and dynamic complexity

The ageing process that encompasses multidimensional changes in physical and psychosocial domains gives rise to high levels of heterogeneity in health status, functioning and healthcare needs [1]. This is particularly relevant to falls and other geriatric syndromes with multifactorial risk profiles spanning the physical, psychological and environmental domains [2, 5, 70]. Heterogeneity introduces variations in falls risk and consequences (e.g., injury severity) and intervention cost, implementation (e.g., care pathways, demand) and efficacy [71]; accounting for these subgroup variations offers opportunities to target subgroups that best meet the decisional criteria (e.g., cost-effectiveness, equity) and/or tailor interventions according to heterogeneous needs [72]. Dynamic complexity arises from intertemporal interaction between causal mechanisms and further increases the heterogeneity between individuals and subgroups. Falls prevention operates in a dynamically complex system characterised by features such as feedback loops (e.g., physical decline increases falls risk and falls accelerate physical decline) (p. 42) [73]. Models should capture the heterogeneity and dynamic complexity of the within-model causal mechanisms—e.g., falls risk, comorbidity level, intervention need and demand—and assess their impact on outcomes.

### Considering theories of human behaviour and implementation

Geriatric health behaviours are strongly shaped by individual psychology (e.g., motivation to prevent functional decline) and social interaction [69, 73–75]. These behaviours determine the prevalence and trajectory of risk factors and the implementation quality of interventions in terms of initial access, adherence and long-term participation [76, 77]. Directly parameterising the psychological and sociological factors would generate model outcomes that are highly sensitive to changes in these factor values [73]. Meanwhile, even just conducting sensitivity/scenario analyses to explore how the model outcomes vary according to different implementation levels would generate useful, heuristic information for decision-makers, specifically regarding investment decisions on auxiliary strategies to improve implementation (e.g., community marketing) [78, 79].

### Considering issues of equity

As highlighted by an international expert panel, healthcare decision-makers face several priority setting criteria beyond cost-effectiveness [54]. These criteria include prioritising the care needs of those in socially deprived subgroups and those with more severe disease and past health loss among similar-age peers. These two vulnerable subgroups overlap in practice given the strong influence of social factors (e.g., income, housing) on health both over the earlier life course and contemporaneously in old age [1, 80]. Prioritising these subgroups likely worsens the overall cost-effectiveness of the intervention given factors such as the ‘double jeopardy’ problem whereby vulnerable groups derive lower efficacy (e.g., due to comorbidity-related contraindications) and/or poorer implementation quality [81, 82]. Models should quantify such equity-efficiency trade-offs and their impact on decisions [37, 82].

## Methods

The systematic review methods were previously described in the aforementioned article [52]. “Systematic review methods” section summarises the associated search methods and inclusion criteria. “Critical appraisal methods” section then describes the methods for critically appraising the included models under the PHMC framework.

### Systematic review methods

The systematic review protocol is registered on the Prospective Register of Systematic Reviews (CRD42021232147). The review followed the 2021 Preferred Reporting Items for Systematic Reviews and Meta-Analyses (PRISMA) guideline [83]: see checklist in separate article [52]. The search covered the period 2003–2020, 12 academic databases and grey literature. A previous systematic review of falls prevention economic evaluations, conducted to inform the NICE falls prevention clinical guideline, covered the period up to 2003 [84]. The current review hence updates the latter, albeit focusing on modelling studies. The search strategy was an intersection between terms for falls, older people, and economic evaluation; the separate article reports all database search strategies [52]. Two researchers independently reviewed the titles and abstracts of identified articles at the first stage and the full texts of approved articles at the second stage.

A study was included if it: (i) targets a population of community-dwelling older persons (aged 60+) and/or individuals aged 50–59 at high falls risk; (ii) evaluates intervention(s) designed to reduce the number of falls or fall-related injuries; (iii) is evaluated against any comparator(s); (iv) reports outcomes of economic evaluation [32]; (v) uses a decision model [32]; and (vi) has English full text. Methodological features of models relevant to the PHMC framework were extracted by JK using a proforma and then discussed in the team regarding their relevance and importance.

### Critical appraisal methods

The methodological features of included models were synthesised by their relevance to the four PHMC challenge categories. Critical appraisal first assessed the prevalence of the given feature across models, then appraised the methods used to incorporate the feature. For example, the proportion of models that accounted for productivity loss associated with falls was assessed. Then, amongst models that accounted for the loss, the range of methods used for doing so were described and compared. The methodological strengths and limitations stated by the models’ authors were used as indicators of desirable modelling practice and scope for improvement, respectively. Where relevant, the methods were also compared against those suggested in the literature informing the PHMC framework in “Challenges to public health economic modelling” section. Based on the appraisal results as well as the literature in “Challenges to public health economic modelling” section, suggestions were made regarding further methodological research in each PHMC challenge category.

## Results

### Search results and overview

In total, 15,730 titles and abstracts and 92 full texts were screened from which 46 decision models were identified. See the separate article for the PRISMA flow diagram and the list of studies excluded at the full-text screening stage [52].

Table 1 provides an overview of the 46 identified models. Most models (n = 25; 52.2%) targeted a general population of community-dwelling adults aged 60+ or 65+. The four types of economic analysis were: cost-effectiveness analysis (CEA); cost–benefit analysis (CBA); return-on-investment analysis (ROI); and cost-utility analysis (CUA), commonly in the form of cost per quality-adjusted life year (QALY). See Additional file 1: Table S1 for definitions of these analysis types. The two costing perspectives were public sector and societal. Some models adopted multiple analysis types and perspectives, resulting in 69 distinct analyses. CUA was most used (n = 32; 46.4%), followed by ROI and CEA (each n = 17; 24.6%), then CBA (n = 3; 4.3%). Around a third of analyses (n = 22) adopted the societal perspective.

**Table 1:** Overview of included decision models for critical appraisal

#	References	Target population	Type of analysis/perspective	Intervention [type] (# of forms)	Model type/time horizon
1	Agartioglu et al. [144]	Fallers aged 65+ admitted to A&E	CEA/PS	HAM	DT/1 year
2	Albert et al. [145]	CD aged 50+ (mean age 75.5)	CUA/PS	MF int.	DT/1 year
3	Alhambra-Borras et al. [146]	CD aged 65+ at high falls risk or frail with no severe physical or cognitive limitation	CUA/PS	Exercise	Markov cohort/lifetime
4	Beard et al. [103]	CD aged 60+	CBA; ROI/PS; Soc	Intersectoral (MC) int.^a^	BD^b^/5 years
5	Boyd et al. [109]	Aged 65+	CUA/PS	Cataract surgery	Markov cohort/20 years
6	Carande-Kulis et al. [97]	CD aged 65+	ROI/US private health insurance	Exercise (2); MC int.	BD/1 year
7	CSP [96]	CD aged 65+	ROI/PS	FRS + exercise [PT]	DT/1 year
8	Church et al. [89]	CD and residential care, aged 65+	CEA; CUA/PS	Exercise (3); MC int.; MF int.; MRA; cataract surgery; Med. mod.; cardiac pacing	Markov cohort/10 years
9	Church et al. [90]	CD aged 65+	CEA; CUA/PS	Exercise (4); MC int.; MF int. (2); MRA; HAM; cataract surgery; Med. mod.; cardiac pacing	Markov cohort/lifetime
10	Comans et al. [105]	CD aged 65+, falls history or gait/functional decline, cognitively intact	ROI/Soc	MF int. (2)	BD/1 year
11	Day et al. [104]	CD, characteristics vary by intervention^c^	CEA/PS; Soc	Exercise (2); HAM; MF int.; Med. mod.; cardiac pacing	DT/1 year
12	Day et al. [93]	CD aged 70+	CEA/PS; Soc	Exercise [Tai Chi]	DT/1 year
13	Deverall et al. [94]	CD aged 65+	CUA/PS; Soc	Exercise (3)	Markov cohort/25 years
14	Eldridge et al. [115]	Aged 65+, CD or nursing home	CUA/PS	FRS + MF int. or exercise	DT + Markov cohort/lifetime
15	Farag et al. [91]	CD aged 65+, no falls history	CUA/PS	Non-specific int.	Markov cohort/lifetime
16	Franklin et al. [92]	CD aged 65+	CUA/PS	FRS + exercise (3) or HAM	DT + Markov cohort/2 years
17	Frick et al. [86]	CD aged 65+	CUA/US healthcare payer^d^	Exercise (2); HAM; MF int. (2); Med. mod.; Vit. D	BD/1 year^e^
18	Hektoen et al. [98]	CD women aged 80+	CEA/Soc	Exercise	BD/1 year
19	Hiligsmann et al. [101]	Aged 60+ with osteoporosis	CUA/Soc	Vit. D and calcium	Markov patient/lifetime
20	Hirst et al. [123]	Women 75+ on pain medication	CUA/PS	Med. mod.	Markov patient/1 year
21	Honkanen et al. [119]	Adults aged 65+, CD at baseline	CUA; ROI/Soc	Hip protectors	Markov cohort/lifetime
22	Howland et al. [87]	CD aged 65+, fall admitted to A&E	ROI/US healthcare payer^d^	MC int	BD/1 year
23	Ippoliti et al. [112]	CD aged 65+, mountainous areas	ROI/PS	MF int.	BD/3 years
24	Johansson et al. [107]	CD aged 65+	CUA/Soc	Intersectoral (MC) int.^f^	Markov cohort/lifetime
25	Lee et al. [147]	CD aged 65–80, no falls history	CBA/PS	Vit. D [targeted vs. universal]	DT + Markov cohort/3 years
26	Ling et al. [88]	CD aged 65+, falls history/risk factors	ROI/US healthcare payer^d^	HAM	BD/1 year
27	McLean et al. [148]	CD aged 70+	CEA; CUA/PS	Exercise	DT/18 months
28	Miller et al. [106]	CD aged 50+ at high falls risk	ROI/US healthcare^d^; Soc	MC int.	BD/2 years
29	Mori et al. [95]	CD women 65+, no osteoporotic fracture	CUA/Soc	Exercise [alone or with bisphosphonate]	DT + Markov patient/lifetime
30	Moriarty et al. [120]	CD aged 65, no adverse events from benzodiazepine/PPI	CUA/PS	Med. mod.	DT + Markov patient/35 years
31	Nshimyu-mukiza et al. [113]	Women aged 40+ (subgroup 65+)	CEA; CUA/PS	Fracture risk screening + physical activity, Vit. D and calcium, and/or Osteoporosis screening and treatment	DT + Markov patient/lifetime
32	OMAS [114]	CD aged 65+	CEA; ROI/PS	Exercise; HAM; Vit. D and calcium; Med. mod.; gait-stabilizer	Markov cohort/lifetime
33	Pega et al. [111]	CD aged 65+	CUA/PS	HAM	Markov cohort/lifetime
34	Poole et al. [149]	Aged 65+	ROI/PS	Vit. D	BD/1 year
35	Poole et al. [150]	CD aged 60+	CUA; ROI/PS	Vit. D	Markov cohort/5 years
36	PHE [116]	CD aged 65+	CUA; ROI/PS	Exercise (3); HAM	DT/2 years
37	RCN [84]	CD aged 60+	CUA/PS	Exercise; MF int.	Markov cohort/lifetime
38	Sach et al. [99]	Women 70+, bilateral cataracts	CEA; CUA/PS; Soc	Cataract surgery [first eye]	BD/lifetime extrapol.^g^
39	Sach et al. [100]	Women 70+, second operable cataract	CUA/PS; Soc	Cataract surgery [second eye]	BD/lifetime extrapol.^g^
40	Smith et al. [85]	Aged 65+ covered by GP and hospital	ROI/PS	FRS + MF int	Risk prediction/1 year
41	Tannenbaum et al. [121]	CD aged 65+ and insomnia	CUA/PS	Med. mod.; CBT	Markov cohort/5 years
42	Turner et al. [124]	CD aged 65+ chronic sedative use for insomnia	CUA/PS	Med. mod.	DT + Markov cohort/1 year
43	van der Velde et al. [151]	CD geriatric outpatients with falls history	CEA/PS	Med. mod.	BD/1 year^e^
44	Wilson et al. [110]	CD aged 65+	CUA/PS	HAM	Markov cohort/lifetime
45	Wu et al. [102]	CD Medicare beneficiaries aged 65+ and falls history	CEA; ROI/PS; Soc	MF int.	BD/1 year
46	Zarca et al. [122]	Aged 65+ without previous hip fracture	CEA; CUA/PS	Vit. D (targeted in two ways vs. universal)	DT + Markov patient/lifetime

*BD* binary decision (model), *CBA* cost–benefit analysis, *CBT* cognitive behavioural therapy, *CD* community-dwelling, *CEA* cost-effectiveness analysis, *CSP* Chartered Society of Physiotherapy, *CUA* cost-utility analysis, *DT* decision tree, *Exp.* Expedited, *FRS* falls risk screening, *HAM* home assessment and modification, *Int.* intervention, *Med. mod.* medication modification, *MC* multiple-component, *MF* multifactorial, *MRA* multifactorial risk assessment only, *OMAS* Ontario Medical Advisory Secretariat, *PHE* Public Health England, *PPI* proton pump inhibitor, *PS* public sector, *PT* physiotherapy, *RCN* Royal College of Nursing, *ROI* return on investment analysis, *Soc* societal

^a^Included individually tailored education, HAM and exercise and public space safety improvement

^b^Binary decision models include two scenarios, with or without intervention, and no time-based cycles or probability trees

^c^Cardiac pacing targeted population aged 50+ due to high falls risk. Other interventions targeted populations aged 65+

^d^This would include public Medicare/aid, private health insurance and patients

^e^1-year horizon with lifetime costs of falls

^f^Included individually tailored education, group balance exercises, Tai Chi, other physical activities and HAM, neighbourhood hazard removal and housing reconstruction

^g^1-year trial outcomes are extrapolated over lifetime horizon

Exercise was the most evaluated intervention type (n = 17 models), followed by multifactorial intervention (n = 13). There were four model type categories: binary decision (n = 14 models)—comparing the state of the world with and without the intervention and without transition probabilities or time cycles; static (n = 9)—all except one [85] were decision trees without time cycles; cohort-level Markov (n = 19); and patient- or individual-level Markov (n = 4). Time horizons varied between 1 year and lifetime; cycle lengths (for models with cycles) varied between 1 month and 1 year.

### Critical appraisal results

The critical appraisal results are presented by the four categories of public health modelling challenges.

#### Capturing non-health outcomes and societal intervention costs

Eighteen models operationalised analyses from the societal perspective (see Table 1). Of these, 15 described the non-health outcomes and/or societal intervention costs incorporated in the analyses, as tabulated in Table 2. The three models not included in Table 2 conducted evaluations from the US healthcare perspective (broader than Medicare/Medicaid), which would have included private healthcare costs, but did not specify the cost type or proportion by sector [86–88].

**Table 2:** Non-health outcomes and societal intervention costs included in models

Study label^a^	Non-health outcomes				Societal intervention costs		
	Social wellbeing	Out-of-pocket expenditure	Productivity	Informal caregiver burden	Social cost	Private co-payment	Time opportunity cost^b^
Beard et al. (2006)						×	Not costed
Carande-Kulis et al. (2015)							×
Comans et al. (2009)						×	
Day et al. (2009, 2010)						×	
Deverall et al. (2018)						×	Not costed
Hektoen et al. (2009)		×					
Hiligsmann et al. (2014)		×					
Honkanen et al. (2006)	×				×		
Johansson et al. (2008)			×	×		×	×
Miller et al. (2011)						×	×
Mori et al. (2017)							×
Sach et al. (2007, 2010)		×		×			
Wu et al. (2010)		Private insurance					

^a^See Table 1 for study references

^b^Time opportunity cost of older participants and volunteers corresponding to productivity. No study incorporated time opportunity cost of informal caregivers attending intervention corresponding to informal caregiver burden; there is hence no dedicated column

Only Honkanen et al. [119] incorporated the social wellbeing loss of fracture-induced residence change from community to nursing home, expressed within a health state utility (HSU) decrement. Here, HSU values are based on a preference-based quality-adjustment scale anchored at 0 (a state equivalent to dead) and 1 (full health) which can be combined with length of life to estimate QALYs; the HSU decrements represent a move away from a more preferred health state. Other models similarly assigned utility decrements for long-term care admission, but it was unclear whether these were specifically associated with residence change or with severity of admission-related fall [89–92]. The use of HSU values to express social wellbeing is noteworthy given their narrow health dimensions. The exclusion of non-health benefits of interventions were frequently mentioned as a limitation, particularly for exercises generating social participation [93–95] and wellbeing gain (e.g., self-confidence) [96, 97]. On the intervention cost side, Honkanen et al. [119] expressed the social cost of hip protector use (discomfort, embarrassment) again within a HSU decrement; the impact was significant, producing an overall QALY loss from intervention for younger subgroups.

Five models incorporated OOP care expenditure: transport costs for fallers [98]; home care [99, 100]; non-specific care [101]; and cost of private insurance [102]. Of these, none incorporated private co-payments as an intervention cost. Seven incorporated intervention co-payments borne by individuals or organisations (but not OOP care expenditure): exercise enrolment [93, 103, 104]; transport cost for participants [105]; venue hire [94, 106]; and local stakeholder involvement [107].

Only Johansson et al. [107] incorporated productivity as an outcome. Specifically, the productivity value (net consumption) was assigned to older persons by age group; hip fractures would then reduce net productivity by shortening life expectancy. On the intervention cost side, Johansson et al. [107] included the time opportunity costs of unpaid volunteering and older persons’ participation. Three further models incorporated time opportunity costs of volunteers and/or participants but not productivity outcomes [95, 97, 106]. Two models mentioned time commitments by volunteers but did not apply monetary values [94, 103].

Three models incorporated informal caregiver burden as productivity loss [99, 100, 107]; none incorporated the health impact on caregivers. Likewise, none incorporated the informal caregivers’ time opportunity cost in accompanying intervention participants.

Two models—both evaluating a combined programme of environmental modifications and multifactorial intervention—discussed the intervention impact on community empowerment but did not quantify it [103, 107]. Both also perceived community involvement—community healthcare staff raising falls risk awareness [103] and local stakeholders designing and delivering interventions [107]—solely as intervention costs rather than as empowerment.

Where outcomes are generated and costs incurred outside the public healthcare system, their valuation methods should change accordingly [32]. Yet only Beard et al. [103] used the value of a statistical life to estimate the *consumption* value of disability-adjusted life year (DALY) burden of falls under CBA. There may also be intersectoral differences in productive efficiencies (i.e., outcome generated per resource invested), leading to intersectoral variations in cost-effectiveness thresholds [108]. Typically, public healthcare systems generate less health per input (i.e., have lower productive efficiency) than what people are willing to pay for and obtain in the private sector (p. 97–98) [32]. Hence, the cost-effectiveness threshold for comparing the incremental benefits and costs under the public sector is lower than that for comparing societal benefits and costs [108]. Incorporating this threshold differential may have changed the final decision in several models [94, 99, 100].

Overall, comprehensive incorporation of non-health outcomes and societal intervention costs is uncommon among models operationalising analyses from the societal perspective. A pressing issue is the balanced incorporation of non-health outcomes and societal intervention costs to prevent models over-estimating (if intervention costs are excluded) or under-estimating (if outcomes excluded) the cost-effectiveness of interventions.

#### Considering heterogeneity and dynamic complexity

##### Heterogeneity

Overall, 27 (58.7%) models conducted at least one analysis related to heterogeneity. Table 3 categorises these analyses into subgroup analysis (SA), targeting analysis (TA), and analysis of heterogeneous intervention needs (IN) and specifies the relevant subgroup delineating variables.

**Table 3:** Methods for assessing heterogeneity in decision models

Study label^a^	Subgroup delineating variables						
	Age	Sex	Social	Falls history	Falls risk^b^	Chronic disease and Med. use	Physical capacity
Alhambra-Borras et al. (2019)	SA	SA					
Boyd et al. (2020)	SA	SA	SA: ethnic	SA			
Carande-Kulis et al. (2015)	TA			TA			
CSP (2016)							
Day et al. (2009)	IN			IN		IN	IN
Deverall et al. (2018)	TA	SA	SA: ethnic				
Eldridge et al. (2005)				IN	IN		
Franklin et al. (2019)	TA						
Hiligsmann et al. (2014)	TA	TA					
Hirst et al. (2016)	TA						
Honkanen et al. (2006)	TA	TA					SA
Ippoliti et al. (2018)			SA: region				
Johansson et al. (2008)	SA	SA					
Lee et al. (2013)	SA	SA					TA
McLean et al. (2015)		SA					
Mori et al. (2017)	TA					TA	
Moriarty et al. (2019)						Separate models^c^	
Nshimyumukiza et al. (2013)	TA					IN	
OMAS (2008)		SA; IN			IN	IN	IN
Pega et al. (2016)	TA	SA	SA: ethnic	TA			
Poole et al. (2014)	SA; TA	SA					
Poole et al. (2015)	SA; TA						
PHE (2018)				IN			
Smith et al. (2016)					TA		
Wilson et al. (2017)	TA	SA	SA: ethnic	TA			
Wu et al. (2010)	SA						
Zarca et al. (2014)	TA	SA					TA

*CSP* Chartered Society of Physiotherapy, *ethnic.* Ethnicity, *Exo* exogenous, *Int.* intervention, *IN* intervention need, *Med.* medication, *OMAS* Ontario Medical Advisory Secretariat, *PHE* Public Health England, *RCN* Royal College of Nursing, *SA* subgroup analysis, *TA* targeting analysis

^a^See Table 1 for study references

^b^Models which evaluated a falls risk screening process and targeted intervention at high fall risk individuals in base case analysis but did not explore alternative non-targeted or differently targeted scenario(s) (e.g., CSP [96] and Franklin et al. [92]) were not marked as having performed TA

^c^The study constructed separate models for non-steroidal anti-inflammatory drug, benzodiazepine and proton pump inhibitor users. The latter could be interpreted as intervention subgroups within the same target population

Age and sex were the most common delineators, used in 24 models. The use of social delineators was infrequent: four models from the same research group used ethnicity [94, 109–111], while one used health authority region [112]. Smith et al. [85] compared different cut-off levels (i.e., targeting scenarios) based on multivariate falls risk estimated from routine care data. Osteoporosis and carotid sinus hypersensitivity were the only chronic disease delineators [95, 104, 113]; psychotropic medication use delineated intervention need in two models [104, 114]. Physical capacity delineators included mobility, functional status and vitamin D level. Five models incorporated heterogeneous intervention subgroups [104, 113–116]; of these, two incorporated non-mutually exclusive interventions [113, 115].

Pega et al. [111] was unique in characterising heterogeneity in efficacy for the same intervention across recipient subgroups: in one scenario analysis, the falls rate ratio of HAM compared to usual care was set to be 0.62 for those at high falls risk and 0.94 for low risk. Studies generally favoured the use of pooled efficacy estimates from meta-analyses: 23 of 35 models using external efficacy sourced meta-analysis estimates; however, pooled estimates can mask heterogeneity in efficacy. With some exceptions [92, 105, 111, 116], there was little effort to discern whether a single or pooled estimate would better reflect the heterogeneity in efficacy**.** Likewise, heterogeneity in intervention cost was poorly modelled: only Honkanen et al. [119] allowed the cost of a hip protector to vary by recipients’ functional status and residence.

An issue in several models concerned how evaluation outcomes were compared across heterogeneously sized target subgroups. Specifically, there was a need to compare both incremental cost-per-unit ratios (e.g., ICER) and aggregate outcomes; an example of the latter is incremental net monetary benefit (INMB) computed by multiplying the total incremental health (e.g., QALY) gain by the cost-effectiveness threshold to obtain its monetary equivalent and subtracting the total incremental cost. An intervention tailored to a specific subgroup may generate a very favourable cost-per-unit ratio but a low aggregate benefit due to the small subgroup size (p. 118–122) [117]. Accordingly, Day et al. [104] compared both the ICER and total falls prevented across six heterogeneously sized subgroups with different intervention needs; here, Otago exercise had the least favourable ICER (although still cost-effective) but the most favourable aggregate impact in terms of the number of hospitalised falls prevented. By contrast, Moriarty et al. [120] estimated the most favourable ICER for modification of inappropriate non-steroidal anti-inflammatory drug (NSAID) relative to benzodiazepine and proton pump inhibitor (PPI) modifications (all three dominated no modification as comparator); however, the prevalence of inappropriate NSAID use was 4.1% compared to 23.6% for PPI. Hence, if aggregate benefits are considered, PPI modification likely becomes the policy priority, not NSAID as concluded by the study. Likewise, PHE [116] estimated that HAM had the most favourable ROI and ICER (with no intervention as comparator) relative to three exercise interventions; but HAM targeted a subgroup 17 times smaller than the latter, thus generating the smallest INMB.

##### Dynamic complexity

Table 4 shows the time-variant falls risk factors and determinants of background health (expressed in HSU values) and comorbidity care costs incorporated in 17 models (13 cohort- and four individual-level Markov models) with time horizons longer than 5 years.

**Table 4:** Time-variant falls risk factors and determinants of background health state utility and care cost progressions in non-binary models with horizons longer than 5 years

Study label^a^	Time-variant falls risk factors			Background^b^ health/cost determinants	
	Age	Falls incidence	Other	Health state utility	Comorbidity care cost
Cohort-level Markov models^c^					
Boyd et al. (2020)	Tunnel state?^d^	MA fall		Age^e^	Age^e^
Church et al. (2011, 2012)	Tunnel state?	Any fall		Age^f^	
Deverall et al. (2018)	Tunnel state?	MA fall		Age^e^	Age^e^
Eldridge et al. (2005)	Tunnel state?	Fracture	FoF	Unclear^f^	Post-fracture^g^
Farag et al. (2015)	Tunnel state?	Any fall		Age^f^	
Honkanen et al. (2006)	Tunnel state?	Hip fracture	FS	Age^e^; FS; LTC; post-hip fracture	FS; LTC
Johansson et al. (2008)	Tunnel state?	Hip fracture		Age^e,f^	Age
Moriarty et al. (2019)	Tunnel state?	MA fall, Hip fracture		Age^f^	
OMAS (2008)	Tunnel state?	MA fall		CEA	
Pega et al. (2016)	Tunnel state?	MA fall		Age^e^	Age^e^
RCN (2005)	Tunnel state?			Age	
Wilson et al. (2017)	Tunnel state?	MA fall		Age^e^	Age^e^
Individual-level Markov models					
Hiligsmann et al. (2014)	×			Post-hip and vertebral fractures	Post-hip fracture^g^
Mori et al. (2017)	×	Fracture	Osteoporosis	Age; post-hip and vertebral fractures	Post-hip fracture^g^
Nshimyumukiza et al. (2013)	×	Fracture	BMD	Post-hip and vertebral fractures	
Zarca et al. (2014)	×	Hip fracture	Vitamin D	Age; post-hip fracture	

*BMD* bone mass density, *CEA* cost-effectiveness analysis, *FoF* fear of falling, *FS* functional status, *LTC* long-term care, *MA fall* fall requiring medical attention, *OMAS* Ontario Medical Advisory Secretariat, *RCN* Royal College of Nursing

^a^See Table 1 for study references

^b^Not directed related to but indirectly influenced by falls/fractures: e.g., fatal fall influences lifetime comorbidity care costs

^c^Alhambra-Borras et al. [146] was excluded due to unclear description of the dynamic model states following intervention

^d^Age-based risk progression would require tunnel states but this was not mentioned or graphed, hence the question mark

^e^Stratified by further time-invariant factors including sex and ethnicity

^f^Unclear whether events such as fracture and LTC admission incurred a one-off or permanent health utility loss

^g^Incorporated ongoing care costs for serious fractures, which are not technically comorbidity care costs since they are directly associated with fall/fracture in model; but they can be interpreted as such given their permanent nature

The time-variant risk factors were grouped into three categories: ‘age’, ‘falls incidence’, and ‘other’ (e.g., fear of falling). For the four individual-level Markov models, fracture risks were updated for individuals by age progression in each cycle. For the 13 cohort-level models, accounting for the age-based risk progression would have required tunnel states for each model state, but this was not mentioned or graphed. Tunnel states exist within each pre-specified Markov model state and have differing transition probabilities to other states to reflect the changes in risk that would occur over the time spent in the given model state [118]; the proportion of the Markov cohort who do not transition to another model state would instead transition to the tunnel states. Lack of tunnel states would bias the results against those who are younger at baseline: the falls risk kept low despite ageing would reduce the absolute number of falls prevented by the intervention and hence its cost-effectiveness. Fifteen incorporated fall/fracture incidence within modelled time as a risk factor, establishing a feedback loop. Only five models incorporated progression of other risk factors. Eldridge et al. [115] modelled individuals transitioning in and out of the state of fear of falling which increased the risks of hip fracture, long-term care (LTC) admission and mortality. Honkanen et al. [119] modelled transitions to functional dependence which increased the risks of hip fracture, LTC admission and mortality. Therefore, Eldridge et al. [115] and Honkanen et al. [119] captured the natural trajectory of geriatric health (using binary indicators) that interacted with fracture incidence and risk.

Only two models incorporated dynamic changes to intervention need: Honkanen et al. [119] allowed the type of hip protector to change according to functional status; Nshimyumukiza et al. [113] shifted individuals to a reactive fracture prevention pathway when a fracture occurred. It was unclear in Nshimyumukiza et al. [113] whether fracture risk screening was repeated each cycle to change the proactive intervention components. Eldridge et al. [115] did not model the progressions of factors included in the falls risk assessment tool (FRAT; e.g., mobility, chronic diseases, medication use) that determined the proactive components nor incorporate repeated risk screening after the baseline year. Deverall [94] was unique in allowing the cost of group exercise to vary dynamically from NZ$480 per person in the 1st year to NZ$62 in subsequent years for those who persist.

All models in Table 4 except OMAS [114] performed CUA. Of these, Honkanen et al. [119] was most thorough in characterising the trajectory of HSU values which progressed by age, functional status, residence and hip fracture incidence. The four individual-level models did not incorporate such geriatric health/frailty progression, but allowed severe fractures to have permanent impacts on utilities. Nine allowed utility progression by age alone, which precluded capturing the heterogeneous health progression within the same age group. Honkanen et al. [119] was again most thorough in characterising the trajectory of comorbidity care costs.

Additional file 1: Table S2 describes the entry and exit patterns for the above non-binary models. Nshimyumukiza et al. [113] was unique in incorporating incoming cohorts each year for the first 10 years. Other models mentioned the non-incorporation of incoming cohorts as a limitation that underestimated the total intervention costs and benefits [93, 104, 111]. Wilson et al. [110] and Pega et al. [111] incorporated annual probabilities of households moving in/out of modified housing which altered the need for HAM. Concerning model exit via mortality, four used fall-related and other-cause mortality rates [94, 109–111], while the rest used fall-related and all-cause rates, which double-counts the former. Seven faced a similar issue in double-counting fall-related LTC admission [89–91, 114, 115, 119, 120].

Overall, the assessment of heterogeneity was limited, confined primarily to comparisons delineated by demographic factors and binary disease or physical capacity status (e.g., mobile vs. non-mobile); the latter neglects the nature of geriatric health best characterised as a position on a continuous spectrum [1]. Likewise, the dynamic progressions in falls risk profile, intervention need, health utilities and care costs were poorly captured.

#### Considering theories of human behaviour and implementation

No model directly parameterised psychological and social causal mechanisms based on individual and social behavioural theories. Nevertheless, 31 (67.4%) models reported at least one implementation level, as shown in Table 5. See also Additional file 1: Table S3 for references concerning the terms used to describe the implementation levels which are distinguished by demand and supply dimensions: e.g., uptake and adoption describe demand and supply for initial access, respectively.

**Table 5:** Summary of base case implementation levels, evidence source and outcomes in associated sensitivity analyses

Study label^a^	Intervention	Base case implementation levels^b^				Sensitivity analysis outcome
		Initial access	Compliance	Sustainability (model time horizon)	Evidence source	
Albert et al. (2016)	MF int.		Adherence: 78.6% Fidelity: 84.1%		Internal non-randomised	No analysis
Alhambra-Borras et al. (2019)	Exercise	Uptake: 39.6%			Internal quasi-experimental	No analysis
Beard et al. (2006)	MC (intersectoral) int.			Maint.: 5 years (of 5)	Internal quasi-experimental	No analysis
Church et al. (2011)	Multiple types			Maint.: 1 year (of 10)	Assumption	No analysis
Church et al. (2012)	Multiple types			Maint.: 1 year (of lifetime)	Assumption	No analysis
Comans et al. (2009)	MF int. (2 forms)	Uptake: as scenario			Assumption	ROI break-even
Day et al. (2009, 2010)	Multiple types^c^	Uptake: 1.9% Tai Chi; 39.4% home exercise; 55.4% HAM; 55.4% MF int.; 18.9% Psychotropic med. withdrawal; 80.0% Cardiac pacing		Persistence: 61% home exercise Maint.: 1 year (of 2) home exercise; 1 year (of 5) cardiac pacing	External RCT	Falls and hospitalised falls averted; ICER (CEA)
Deverall et al. (2018)	Group (commercial) exercise	Uptake: 52%			External RCT	Inc. cost; Inc. QALY; ICER (CUA)
				Persistence: 80.5% uptake in year 2; 10% in year 10	External RCTs and assumption	Same as uptake
				Maint.: permanent	External RCT	No analysis
	Home exercise	Uptake: 52%			External RCT	Inc. cost; Inc. QALY; ICER (CUA)
				Persistence: 76.3% uptake in year 2; 10% in year 5	External RCTs and assumption	Same as uptake
				Maint.: permanent	External RCT	No analysis
Eldridge et al. (2005)	FRS + MF int. or exercise (prescribed or self-referred)	Uptake: 6.5% FRS; 50%/10% self-referred exercise for high-/low-risk persons			Internal survey	Proportion of total falls averted
Farag et al. (2015)	Non-specific intervention	Uptake: 50%			Assumption	ICER (CUA)
Franklin et al. (2019)	FRS + exercise (3 forms) or HAM	Uptake: 100% for those referred from FRS			Assumption	ICER (CUA)
				Maint.: 1 year (of 2)	Assumption	No analysis
Hiligsmann et al. (2014)	Vit D and calcium supplement			Maint.: 3 years (of lifetime)	Assumption	ICER (CUA)
Hirst et al. (2016)	Med. modification		Adherence: 29.4% of eligible days		External survey	Inc. cost; Inc. QALY; ICER (CUA)
Honkanen et al. (2006)	Hip protector		Adherence: 36% of daily hours		External survey	ICER (CUA)
				Persistence: 50% discontinue after 1st year; discontinuation rate declines exponentially	External survey	ICER (CUA)
Howland et al. (2015)	MC int. (lay-led)	Uptake: 50%			Assumption	Aggregate efficiency (ROI: net cost saving)
			Fidelity: 100% refer		Assumption	No analysis
Ippoliti et al. (2018)	MF int.	Uptake: 80%			Assumption	No analysis
Johansson et al. (2008)	MF int.			Maint.: 5 years (of lifetime)	Internal quasi-experiment	No analysis
Lee et al. (2013)	Vit D screening and supplement		Adherence: 80%		External RCT	No analysis
Miller et al. (2011)	MC int. (lay-led)		Adherence: 71.4%	Maint.: 1 year (of 2)	Assumption	No analysis
Mori et al. (2017)	Home exercise	Uptake: 42%			External RCTs	No analysis
				Maint.: 1 year (of lifetime)	Assumption	Inc. cost; Inc. QALY; ICER (CUA)
Moriarty et al. (2019)	Med. modification (Benzodiazepine, PPI)		Adherence: 100%		Assumption	Inc. cost; Inc. QALY
Nshimyumukiza et al. (2013)	Fracture risk screening + physical activity, Vit D and calcium, and/or Osteoporosis screen and treatment	Uptake: 53%			External survey	ICER (CEA, CUA)
				Maint.: permanent	Assumption	No analysis
OMAS (2008)	Multiple types	Uptake: 57.0% exercise; 27.0% psychotropic med.; not specified for HAM, Vit D, Gait stabiliser	Adherence: 79.0% exercise; 75.7% HAM; 81.8% Vit D; 53.0% psychotropic med.; 80.0% Gait stabiliser		External RCTs and survey	No analysis
				Persistence: same as adherence	Assumption	No analysis
Pega et al. (2016); Wilson et al. (2017)	HAM	Uptake: 89.0%			External RCT	Inc. cost; Inc. QALY; ICER (CUA)
				Maint.: one-off, no renewal	Assumption	No analysis
Poole et al. (2015)	Vit D supplement			Maint.: 5 years (of 5)	External RCTs	No analysis
PHE (2018)	Exercise (3 forms); HAM	Uptake: 20%		Maint.: 1 year (of 2)	Assumption	No analysis
Turner et al. (2020)	Med. modification	Adoption: 66% of GPs received pharmacist advice; 79% met older persons for deprescribing			Unclear^d^	Inc. cost; Inc. QALY; ICER (CUA)
		Uptake: 53%			External RCT	No analysis
Wu et al. (2010)	MF int.	Uptake: 50%			External RCT and surveys	Aggregate efficiency (ROI: net cost saving); ICER (CEA)
Zarca et al. (2014)	Vit D screening and supplement		Adherence: 50%; 100% after fracture		External survey and assumption	ICER (CUA)
				Maint.: permanent	Assumption	No analysis

*CBT* cognitive behavioural therapy, *CEA* cost-effectiveness analysis, *CSP* Chartered Society of Physiotherapy, *CUA* cost-utility analysis, *FRID* fall risk increasing drug, *FRS* falls risk screening, *HAM* home assessment and modification, *ICER* incremental cost-effectiveness ratio, *Inc.* incremental, *Int.* intervention, *Maint.* Maintenance, *MC* multiple-component, *MF* multifactorial, *OMAS* Ontario Medical Advisory Secretariat, *PHE* Public Health England, *PPI* proton pump inhibitor, *QALY* quality-adjusted life year, *RCT* randomised controlled trial, *ROI* return on investment

^a^See Table 1 for study references

^b^Supply and demand dimensions to implementation levels are distinguished: uptake (demand) and adoption (supply) for initial access; adherence and fidelity for compliance; and persistence and maintenance for sustainability. See Additional file 1: Table S3 for the references concerning the terms used

^c^The configuration is the same for Tai Chi in Day et al. [93]

^d^Cites the model Moriarty et al. [120] which does not report the parameter estimates directly

A notable feature was the frequent reliance on modeller assumptions to parameterise the implementation levels, which was widely acknowledged as a limitation by authors [96, 97, 104, 106, 113, 121]. Of the 18 models that reported access levels, five relied on assumptions [87, 91, 92, 112, 116]. Only Turner et al. [124] distinguished between adoption and uptake: in the main intervention scenario, professionals’ adoption of sedative de-prescribing is imperfect, and only in an alternative scenario does it become 100%; meanwhile, older persons’ uptake remains at 53% in both scenarios. Nine models reported compliance levels, four relying on assumptions [87, 106, 120, 122]. Honkanen et al. [119] uniquely applied per-protocol rather than intention-to-treat (ITT) efficacy from RCT; the adherence rate then determined the intervention effectiveness. In other models that used ITT evidence *and* applied compliance rates, there was a risk of confounding. For example, OMAS [114] specified the adherence rates for their interventions and seemingly applied the ITT efficacies to both adherers and non-adherers, which would underestimate the efficacy for adherers and overestimate for non-adherers. Of the 19 models that reported sustainability durations, 13 used assumptions [89, 90, 92, 95, 101, 106, 107, 110, 111, 113, 114, 116, 122]. Under long model horizons, rudimentary assumptions on intervention sustainability would produce misleading results. For example, Church et al. [90] did not allow for sustained access to ongoing interventions such as exercise, while one-off procedures such as expedited cataract surgery were assumed to generate permanent efficacy, thus significantly advantaging the latter.

Table 5 also lists the outcomes used for one-way sensitivity or scenario analyses that involved changes in implementation levels. Twelve of 31 (38.7%) models did not assess varying implementation levels, and several acknowledged this as a limitation [92, 101, 112]. Cost-per-unit ratios were the most common outcomes used (by 15 models). A key disadvantage of ratios is that their association with implementation levels depends strongly on the cost summary method, specifically whether fixed/sunk intervention costs are incorporated. If fixed costs are translated to per-participant rates, higher implementation level would raise the net intervention cost and health benefit at the constant rates such that the cost-per-unit ratio remains constant. Accordingly, models without fixed costs found that varying implementation had minimal impacts on cost-per-unit ratio [91, 93–95, 102, 104, 110, 111, 119]. By contrast, Comans et al. [105] incorporated fixed costs and estimated the uptake rates needed for multifactorial interventions to generate ROI ratios above one. Hence, ratios ought to be interpreted alongside aggregate outcomes to enable holistic evaluations. For example, Zarca et al. [122] found that adherence rate of 30% was sufficient to generate a favourable ICER and ruled out further information campaigns to improve adherence; but this potentially neglects the aggregate benefits foregone by low adherence.

Twelve models evaluated the effect of varying implementation on aggregate outcomes: total number of falls prevented [93, 104, 115]; incremental costs and QALYs in CUA [94, 95, 110, 111, 120, 123, 124]; and aggregate net economic saving in ROI [87, 102]. Of the latter, Howland et al. [87] estimated that increasing the uptake of multiple-component interventions from a base case rate of 50% to 75% would generate savings of $2.79 million for a population of 44,000. This represents the maximum amount the decision-maker could spend on auxiliary implementation strategies to achieve the bespoke uptake increase. Yet, models seldom discussed *how* the variations in implementation levels were generated, often assessing the variations under deterministic sensitivity analysis (i.e., to assess parameter uncertainty) rather than under scenario analysis as distinct intervention strategies (e.g., [94, 110, 111]).

An issue related to implementation is the presence of capacity or budget constraint which defines the feasible levels of implementation [72]; no model incorporated such constraints. PHE [116], for example, assumed that in a typical English local health authority area, around 5000 older persons would receive group exercise at any time. With a maximum of 10 participants per group, this would require 500 venues per week, which is likely beyond the venue capacity of most local health authorities. For decision-makers overseeing a specific geographical region, the sizes of the newly incoming cohorts (e.g., newly turned age 60 or 65) would affect the capacity use over time; yet, as mentioned, only Nshimyumukiza et al. [113] incorporated incoming cohorts (and only for the first 10 years).

Overall, the most pressing methodological issue regarding behaviour and implementation concerns parameterising the long-term intervention sustainability using appropriate evidence rather than modeller assumptions. Moreover, there is greater scope for conducting value of implementation (VoIM) analyses and incorporating capacity constraints to improve model credibility [72, 78].

#### Considering issues of equity

Only seven (15.2%) models, shown in Table 6, incorporated vulnerable subgroups based on social deprivation and/or health severity. Two potential causes of reduced capacity to benefit for these subgroups (relative to non-vulnerable peers) could be discerned from the models: (i) the double jeopardy (DJ) problem—i.e., vulnerable individuals derive lower efficacy and/or poorer implementation quality; and (ii) life expectancy differential (LED) problem—i.e., vulnerable individuals derive less QALY gain from an intervention improving HSU values due to shorter remaining life expectancy.

**Table 6:** Social and severity subgroups in models and cause(s) of reduced capacity to benefit

Study label^a^	Subgroup delineator^b^	Cause of reduced capacity to benefit	Note
Four BODE3 models^c^	Social: ethnicity (Maori vs. non-Maori)	Life expectancy differential	Also explores outcome differences across sex subgroups. Difficult to explore the double jeopardy problem due to homogenous intervention efficacy, cost and implementation level across subgroups
Eldridge et al. (2005)	Severity: fear of falling	Life expectancy differential	Fear of falling experienced by fallers and non-fallers and hence can be interpreted as frailty. Does not report subgroup results
Honkanen et al. (2006)	Severity: functional dependence	Double jeopardy; life expectancy differential	
Smith et al. (2016)	Severity: multivariate falls risk/frailty^d^ profile	No reduced capacity	Multivariate falls risk/frailty profile contained age, sex, all-cause secondary care use, fall and fracture history, chronic diseases and polypharmacy; all persons had same intervention efficacy, cost and implementation level

*BODE3* Burden of Disease Epidemiology, Equity & Cost-Effectiveness, *HAM* home assessment and modification

^a^See Table 1 for study references

^b^In identifying health severity subgroups, age, sex and individual fall/fracture risk factors (e.g., falls history, mobility impairment, bone mass density, vitamin D level, psychotropic medication use) were not interpreted as delineators. Studies that targeted specific patient groups within general older populations were excluded

^c^Deverall et al. [94], Boyd et al. [109], Wilson et al. [110] and Pega et al. [111] which were developed by the Burden of Disease Epidemiology, Equity and Cost-Effectiveness (BODE3) research group in New Zealand

^d^The falls risk prediction tool included several indicators of generalised frailty (e.g., recurrent secondary care utilisations)

Only the four models from the same BODE3 research group incorporated social subgroups delineated by ethnicity (Maori vs. non-Maori) [94, 109–111]. All four reported lower QALY gains and higher ICERs for the Maori group. Except Boyd et al. [109], they also investigated the cause of reduced capacity to benefit: under a hypothetical scenario of equal life expectancy, the Maori group experienced *higher* per-capita QALY gain than the non-Maori group. Hence, the LED problem was identified as the main cause of reduced capacity. But homogenous parameters across ethnic subgroups for intervention cost, efficacy and implementation precluded analysis of the DJ problem. The latter was only narratively discussed, with reference to non-ethnic social delineators. For example, Wilson et al. [110] mentioned that HAM uptake is likely lower for low-income populations who are likelier to rent; Pega et al. [111] mentioned that public campaign to promote do-it-yourself HAM would disproportionately benefit homeowners.

Three models incorporated severity subgroups delineated by variables other than age, sex and individual falls risk factors (e.g., falls history). Eldridge et al. [115] incorporated a severity subgroup of individuals with fear of falling as indicator of generalised frailty. Fear did not influence the intervention type, efficacy, cost or uptake; hence, the DJ problem was precluded. Instead, the higher mortality risk faced by those with fear means that the LED problem was present. Another key feature is the model’s incorporation of health utility levels for health states: those with and without fear had utilities of 0.67 and 1, respectively, prior to fracture, and the same utility 0.31 after fracture; the respective utility decrements are hence 0.36 and 0.69. This improves the cost-effectiveness of fracture prevention for those *without* fear. Nevertheless, the model did not report any subgroup results.

Honkanen et al. [119] incorporated a severity subgroup of functionally dependent individuals who generated less favourable cost-effectiveness result than the whole population. Specifically, hip protector use no longer dominated no intervention for functionally dependent women aged 80 and 85 unlike the whole population. This could partly be attributed to the DJ problem because the dependent subgroup incurred a higher intervention cost to achieve the same efficacy as the independent. Interestingly, fracture cost was *lower* for the dependent, meaning that fracture prevention is *less* cost-effective for them. The LED likely also contributed via the higher mortality risk faced by the dependent.

Smith et al. [85] operationalised a multivariate falls risk prediction tool that included several indicators of frailty. The 1-year horizon precluded any LED problem. The model applied homogenous intervention efficacy, cost and implementation level for all frailty/risk level, removing any DJ problem. This generated potentially misleading outcomes: for example, the model estimated that only the 1.8% highest risk individuals should be referred to the intervention to achieve positive financial returns; but these individuals likely have significantly reduced capacity to benefit due to their comorbidities. The model hence likely overestimated the cost-effectiveness of targeting the most vulnerable.

No model conducted analyses that quantified the equity-efficiency trade-off. Except Boyd et al. [109], the BODE3 models only explored the hypothetical scenario of equal life expectancy across subgroups and did not evaluate intervention strategies prioritising intervention access/outcomes for the Maori subgroup. They nevertheless discussed several such equity-oriented intervention strategies: Wilson et al. [110] and Pega et al. [111] suggested analysis of HAM targeting low-income renters; Boyd et al. [109] discussed how public sector provision of cataract surgery should counteract worsening health inequity under private sector provision. They did not discuss any methodological aspects of equity analysis such as estimating the relative importance of equity and efficiency gains. For severity-based equity issues, only Honkanen et al. [119] reported the subgroup results but did not evaluate any strategy prioritising the severity subgroup. Overall, equity considerations are limited within existing models.

### Suggestions for further methodological research

The results of the critical appraisal of the models by the systematic review were translated to 16 suggestions for further methodological research, sub-categorised by the four key challenges. These are shown in Table 7 alongside the textual justification for each suggestion.

**Table 7:** Suggestions for further methodological research and justification

Methodological research suggestion	Justification
Challenge 1—Capturing non-health outcomes and societal intervention costs	
1. Explore methods for consulting stakeholders on the appropriate perspective to take (e.g., public sector, societal) and the range of appropriate outcomes and costs, particularly under the societal perspective	Models operationalising the societal perspective were generally limited in terms of the range of societal outcomes and costs incorporated; see Table 2 and “Capturing non-health outcomes and societal intervention costs” section. The appropriate perspective for the evaluation would depend on the range of outcomes prioritised by decision stakeholders [39]
2. Explore methods and data sources for incorporating balanced sets of outcomes and intervention costs under the societal perspective	Balanced incorporation of non-health outcomes and societal intervention costs was achieved by only two of 15 models shown in Table 2; see “Capturing non-health outcomes and societal intervention costs” section. Imbalanced incorporation would risk over- or under-estimating the cost-effectiveness of the intervention
3. Explore methods to account for sector-specific productive efficiencies under the societal perspective and to assess the relevance of established/possible cost-effectiveness thresholds [108]	Accounting for the intersectoral differences in cost-effectiveness thresholds (i.e., productive efficiencies) would have changed the final decision in several models [94, 99, 100]
Challenge 2—Considering heterogeneity and dynamic complexity	
1. Explore methods and data sources for incorporating variables that depict geriatric health and falls risk variations within the same age and sex groups and over time, such as the continuous, multivariate frailty index [152]	Models were limited in terms of incorporating subgroup delineators beyond age and sex (Table 3) and time-variant falls risk factors beyond age and falls history (Table 4). The multivariate frailty index is suggested as a variable that can capture the multidimensional nature of changes to geriatric health and falls risk
2. Explore the impact on intervention rankings of the choice in the main decision metric between cost-per-unit ratio and aggregate outcome [104]	Several models evaluated interventions targeting heterogeneously sized subgroups then compared the resulting ICERs only. This may have introduced misleading interpretations of economic outcomes: see the last paragraph of “Heterogeneity” section
3. Explore methods and data sources for characterising the heterogeneity in intervention efficacy, cost and implementation level	Heterogeneities in intervention efficacy and cost were characterised by only one model each (“Heterogeneity” section, 3rd paragraph). Heterogeneities in intervention access, compliance, and sustainability were likewise highly limited (Table 5)
4. Explore the feasibility of developing individual-level simulation to capture the age-related progression in falls risk and other dynamic patterns in geriatric health aspects (e.g., progressions in comorbidity care costs)	Tunnel states were not described for the 13 cohort-level Markov models in Table 4. Individual-level models are likely better suited to characterise the age-related falls risk progression. Health utilities and comorbidity costs progressed by age groups only. Here again, individual-level simulation can capture the annual progressions and variations by geriatric health variables (e.g., frailty, functional status)
5. Explore methods for modelling: (i) periodic falls risk screening to allow dynamic variation in the proactive intervention pathway (5); and (ii) access to reactive pathway after a serious falls incidence	No model incorporated repeated falls/fracture risk screening to reassess the need for proactive intervention access. Only one model shifted individuals to the reactive pathway once a fracture occurred (“Dynamic complexity” section, 3rd paragraph)
6. Explore methods for modelling incoming cohorts of newly eligible persons to characterise the dynamic target population size and capacity implications	Models mentioned the non-incorporation of incoming cohorts as a limitation that underestimated the total intervention costs and benefits (“Dynamic complexity” section, 5th paragraph). No model considered capacity implication, most affected by incoming cohorts who generate sustained intervention need (“Considering theories of human behaviour and implementation” section, 5th paragraph)
Challenge 3—Considering theories of human behaviour and implementation	
1. Explore methods and data sources for incorporating individual- and social-level variables that influence health behaviour and intervention supply/demand	No model directly parameterised psychological and social causal mechanisms based on individual and social behavioural theories (“Considering theories of human behaviour and implementation” section, 1st paragraph). In Table 5, only one model characterised the long-term variation in demand persistence
2. Explore methods for distinguishing between supply- and demand-side implementation factors and evidence sources for long-term sustainability of interventions	Only Turner et al. [124] distinguished between demand-side uptake and supply-side adoption as determinants of initial access (“Considering theories of human behaviour and implementation” section, 2nd paragraph). Sustainability parameters relied extensively on assumptions (Table 5; “Considering theories of human behaviour and implementation” section, 2nd paragraph)
3. Explore the feasibility of conducting value of implementation analyses as alternative scenarios of implementation strategies with aggregate monetary outcomes to estimate the willingness to pay	Models often assessed the variations in implementation levels under DSA (i.e., to assess parameter uncertainty) rather than scenario analysis (“Considering theories of human behaviour and implementation” section, 3rd paragraph). Cost-per-unit ratios may poorly indicate the impact of implementation change (“Considering theories of human behaviour and implementation” section, 3rd paragraph)
4. Explore the feasibility of developing models that explicitly incorporate capacity constraints, such as discrete events simulation [153]	No model considered the capacity or budget implications of their interventions. This resulted in misleading outcomes; an example is given in “Considering theories of human behaviour and implementation” section, 5th paragraph
Challenge 4—Considering issues of equity	
1. Explore methods for consulting stakeholders to identify relevant social and health severity delineators	Table 6 shows that few models incorporated vulnerable subgroups; ethnicity was the only social delineator of equity relevance. Consulting the stakeholders is facilitates equity analyses relevant to the specific settings (“Considering issues of equity” section) [39]
2. Explore methods and data sources for modelling causal mechanisms behind vulnerable subgroups’ reduced capacity to benefit	Models in Table 6 did not fully account for causal mechanisms of reduced capacity to benefit: e.g., BODE3 models did not parameterise heterogeneous intervention efficacy and access by ethnicity, precluding analyses of the double jeopardy problem (“Considering issues of equity” section, 2nd paragraph)
3. Explore methods for equity analysis that assesses the equity-efficiency trade-off under alternative intervention strategies, such as DCEA [82]	No model evaluated alternative strategies that prioritised the vulnerable subgroups and then estimated the efficiency-equity trade-off (“Considering issues of equity” section, 6th paragraph)

*DCEA* distributional cost-effectiveness analysis, *DSA* deterministic sensitivity analysis, *ICER* incremental cost-effectiveness ratio, *INMB* incremental net monetary benefit, *PHMC* public health modelling challenge, *ROI* return on investment

## Discussion

This systematic review and critical appraisal explored how 46 existing economic models of community-based falls prevention addressed four key methodological challenges associated with public health economic modelling and made methodological research suggestions that may inform future model development. The appraisal results supplement those concerning falls epidemiology, falls prevention intervention, and evaluation methods in the first part of the review results [52].

Although the four challenges were appraised separately, the significant interactions between them should be noted. Incorporation of non-health outcomes would increase the heterogeneity and dynamic complexity in analysis [1]. Wider intervention benefits and costs are closely associated with implementation quality: for example, social benefits act as uptake facilitators [5], and co-payments as barriers [46]. Likewise, long-term behavioural and implementation patterns are dynamically complex due to feedback loops (e.g., initially successful adherence reinforces persistence). Intervention need is also dynamically complex, dependent on history of previous intervention receipts; however, there is little guidance as to how intervention prescription should vary by history [5, 27, 125].

The interactions between the first three challenges and the last (i.e., equity considerations), warrant further attention. First, capturing non-health outcomes and societal intervention costs likely exacerbates the inequitable outcome differences between social- and severity-based subgroups. For example, incorporating wider consumption benefits of HAM advantages richer homeowners [110]. Incorporating productivity loss likely disadvantages the socially deprived and frail subgroups who are less likely to be in paid/unpaid employment prior to a fall. Yet excluding the wider outcomes simply masks the equity consequences (e.g., that publicly funded HAM may constitute a regressive wealth transfer towards homeowners); their inclusion is necessary to design policies that address the holistic needs of vulnerable older persons [126]. The main implication is that evaluations from the societal perspective should rigorously plan equity analyses, engaging with stakeholders both inside and outside the healthcare system to understand intersectoral equity-related priorities.

Second, there is a close overlap between considerations of heterogeneous intervention need and severity-based equity issues [85, 115, 119]. One factor influencing both intervention need and health severity is cognitive impairment, which is a key falls risk factor [5] and a frailty indicator [28]. Yet there is relatively little trial-based efficacy evidence for cognitively impaired persons, with many RCTs purposefully excluding them [29]; they also require tailored intervention attributes such as caregiver accompaniment [66]. These features likely mean that the cognitively impaired experience a substantially different intervention pathway, though there is little guidance in current guidelines [5, 27, 125], as well as a significant DJ problem. Any model targeting the general community-dwelling older population should actively incorporate the dual consideration of intervention need and severity-based inequity since it implicitly targets a sizeable cognitively impaired subgroup; e.g., 22% of UK men aged 65–84 have mild cognitive impairment [127]. Generalised frailty similarly warrants the dual consideration: tailored interventions exist that can generate positive health benefits for the frailest [128]; yet their outcomes are likely markedly worse than those of the less frail subgroups, as shown in one trial-based economic evaluation of multifactorial intervention [81]. Therefore, decision-makers aiming to implement an inclusive intervention programme benefitting the frailest should articulate their severity-based priorities beyond cost-effectiveness [54].

Third, social status and culture are known to influence geriatric health behaviour [129], and health severity can both motivate and deter intervention participation [74, 130]; there is hence another intersection between equity and behavioural and implementation considerations. Complex interventions such as proactive multifactorial intervention may face greater implementation challenges (e.g., routine risk screening, coordinating multidisciplinary team) but better reach vulnerable groups who are less likely to self-refer to voluntary programmes [131]. The consideration of aggregate outcomes in VoIM magnifies the priority setting challenges: vulnerable subgroups forming a minority would likely generate lower aggregate benefits from implementation improvements than the less vulnerable majority (even if the former’s cost-per-unit ratio is more favourable). Indeed, previous applications of the equity-oriented distributional cost-effectiveness analysis (DCEA) framework have compared alternative implementation strategies with differing impacts on efficiency and equity [82, 111, 132]. Conduct of VoIM therefore warrants equity analyses, consulting with stakeholders on the likely subgroup-specific impacts of local implementation strategies.

Consideration of the above modelling challenges holds relevance to other public health areas. Inclusion of non-health outcomes and societal intervention costs has been identified as a key methodological challenge for all geriatric public health interventions [50, 55]. Modelling for other geriatric syndromes that are symptoms of multiple age-related impairments would benefit from consideration of heterogeneity and dynamic complexity [2, 133]. Periodic risk screening to track the dynamic progression in risk and associated intervention changes is relevant to all adult age groups [134]. Diverse integrated care schemes face implementation problems that would benefit from VoIM analyses [135]. Social determinants of health inequities are present over the life course [136, 137], while consideration of disease severity is recommended for all NICE decision-making in the UK [138]. In all, any public health conceptual modelling would benefit from a systematic appraisal of existing models as conducted here for community-based falls prevention [39].

An important avenue of further research concerns the availability of data required to implement the methodological aspects highlighted by the appraisal. Forthcoming work by the current authors seeks to develop a falls prevention economic model that implements the aspects using publicly available data [139], specifically the English Longitudinal Survey of Ageing (ELSA) [140] and falls prevention RCTs. Other longitudinal surveys and electronic health records similarly contain sufficient data to explore the associations between falls, multivariate frailty, and further indicators of geriatric health [42, 141, 142]. Falls prevention RCTs can provide several key data parameters including older persons’ capability and accompanying caregivers’ health [66] and the probability of meeting physical activity targets [143]. Overall, data availability appears not to be an insurmountable obstacle, while any data issues confronted could motivate and inform further primary data collections.

A key strength of this critical appraisal is the range of the methodological challenges covered: one previous systematic review covered only the challenge of capturing non-health outcomes [50], while another covered only equity issues [51]. The range was moreover informed by a previous systematic methodological review [40], supplemented by geriatric health literature (e.g., [1, 2]). A key limitation was the appraisal’s reliance on published content; contacting modellers would have rectified methodological ambiguities in several areas. A further limitation was that the search strategy had not been validated by an expert systematic reviewer or information specialist. Nevertheless, the review achieved the most comprehensive coverage of community-based falls prevention models to date, including 26 models unidentified by previous systematic reviews in this area [49].

An important caveat is that the methodological appraisals in this article do not exhaust the range of issues relevant to falls prevention modelling but rather focus on those highlighted by the systematic methodological review as being critical for public health economic model’s credibility [40]. Further appraisal results reported in the separate article should be referred [52]. Moreover, the methodological research suggestions in Table 7 do not constitute an exhaustive and authoritative list but rather contributes to ongoing methodological discussions. Finally, the broad range of methodological features appraised meant that in-depth discussions around any specific feature were precluded; for these, the literature cited in “Challenges to public health economic modelling” section—e.g., the overview on capturing the health and wellbeing impacts on informal caregivers [61]—should be referred as starting points.

## Conclusion

Existing models for economic evaluation of community-based falls preventions contain methodological limitations spanning four challenge areas relevant for public health economic modelling. The appraisal in this work can inform the conceptual modelling of future falls prevention economic models to increase their credibility, as well as highlighting aspects for further methodological research within public health economic modelling. Stakeholders for modelling should explore how the four challenges and their interactions are specifically relevant to their decision-making context.

## Supplementary Information

**Additional file 1: Table S1.** Definitions of analysis types in economic evaluation. **Table S2.** Dynamic entry and exit patterns for non-binary models with horizons longer than 5 years. **Table S3.** Implementation levels by demand and supply dimensions.

## Data Availability

The extracted data from the systematic review are available from the corresponding author upon reasonable request.

## Abbreviations

CBA: Cost–benefit analysis
CEA: Cost-effectiveness analysis
CG: Clinical guideline
CUA: Cost-utility analysis
DALY: Disability-adjusted life year
DCEA: Distributional cost-effectiveness analysis
DJ: Double jeopardy
HAM: Home assessment and modification
ICER: Incremental cost-effectiveness ratio
INMB: Incremental net monetary benefit
ITT: Intention-to-treat
LED: Life expectancy differential
LTC: Long-term care
NICE: National Institute for Health and Care Excellence
NSAID: Non-steroidal anti-inflammatory drug
OOP: Out-of-pocket
PPI: Proton pump inhibitor
PRISMA: Preferred Reporting Items for Systematic Reviews and Meta-Analyses#
QALY: Quality-adjusted life year
ROI: Return on investment
VoIM: Value of implementation
