# Supplementary material for: Systematic review and critical methodological appraisal of community-based falls prevention economic models
Source: Cost Eff Resour Alloc. 2022 Jul 16;20:33. doi: 10.1186/s12962-022-00367-y (PMC9287934; doi:10.1186/s12962-022-00367-y)
Supplement: Supplementary file 1 — Additional file 1: Table S1. Definitions of analysis types in economic evaluation. Table S2. Dynamic entry and exit patterns for non-binary models with horizons longer than 5 years. Table S3. Implementation levels by demand and supply dimensions. [file 12962_2022_367_MOESM1_ESM.docx]

# Systematic review and critical methodological appraisal of community-based falls prevention economic models

**Additional file 1**

**Authors:**

Mr Joseph Kwon^1*^, [jkwon6@sheffield.ac.uk](mailto:jkwon6@sheffield.ac.uk); ORCID 0000-0002-2860-7280

Dr Hazel Squires^1^, [h.squires@sheffield.ac.uk](mailto:h.squires@sheffield.ac.uk); ORCID 0000-0001-8467-0471

Dr Matthew Franklin^1^, [matt.franklin@sheffield.ac.uk](mailto:matt.franklin@sheffield.ac.uk); ORCID 0000-0002-2776-4014

Professor Tracey Young^1^, [t.a.young@sheffield.ac.uk](mailto:t.a.young@sheffield.ac.uk); ORCID 0000-0002-0754-7223

^1^ School of Health and Related Research, University of Sheffield, Regent Court (ScHARR), 30 Regent Street, Sheffield, England, S1 4DA

* Corresponding author

**Competing interests:** There are no competing interests to declare.

**Funding:** Mr Joseph Kwon was supported by the Wellcome Trust [108903/B/15/Z]

**Authors’ contributions:** All authors were involved in study conceptualisation. JK wrote the first manuscript draft. HS, MF and TY were involved in the writing of subsequent drafts. All authors read and approved the final manuscript.

**Availability of data and materials:** The extracted data from the systematic review are available from the corresponding author upon reasonable request.

**Keywords:** geriatric public health; falls prevention; decision modelling; economic evaluation

# Definitions of analysis types

| **Table S1** Definitions of analysis types in economic evaluation | |
| --- | --- |
| Cost-benefit analysis (CBA) | All costs are measured and valued in monetary units. Health benefits are valued by individuals’ willingness to pay (i.e., consumption value of health) and thus expressed in monetary units. The final evaluation outcome is typically the ratio between incremental monetary benefits and incremental monetary costs of the intervention relative to its comparator. |
| Cost-effectiveness analysis (CEA) | All costs are measured and valued in monetary units. Natural health unit (e.g., the number of falls avoided) is used as the health outcome measure. The final evaluation outcome is typically the ratio of incremental cost per health unit gain of the intervention relative to its comparator. This ratio is compared against a cost-effectiveness threshold (see CUA below). |
| Cost-utility analysis (CUA) | All costs are measured and valued in monetary units. Generic measure of health gain, typically quality-adjusted life year (QALY) gain, is used as the health outcome measure. The final evaluation outcome is typically the ratio of incremental cost per QALY gain of the intervention relative to its comparator. This ratio is compared against a cost-effectiveness threshold that expresses the productive efficiency of the healthcare system in the given decision-making context: i.e., the monetary value of healthcare resources required to generate an additional QALY gain. A ratio below the threshold means that the intervention is cost-effective relative to its comparator. |
| Return on investment (ROI) | All costs are measured and valued in monetary units. No health outcomes are included in the analysis. ROI compares the intervention cost and the total economic savings (e.g., from health and social care utilisations prevented) and then expresses the difference between intervention and comparator in net cost or ratio. |

# Dynamic entry and exit patterns

| **Table S2** Dynamic entry and exit patterns for non-binary models with horizons longer than five years | | | | | | |
| --- | --- | --- | --- | --- | --- | --- |
| **Study label^1^** | **Population** | | **Mortality** | | **LTC admission** | |
|  | Entry | Migration | Fatal fall / Excess mortality | Non-fall-related | Fall-related | Non-fall-related |
| Boyd (2020) |  |  | ˟ | ˟ |  | ˟ |
| Church (2011); (2012) |  |  | ˟ | ˟ | ˟ | ˟ |
| Deverall (2018) |  |  | ˟ | ˟ |  | ˟ |
| Eldridge (2005) |  |  | ˟ | ˟ | ˟ | ˟ |
| Farag (2015) |  |  | ˟ | ˟ | ˟ | ˟ |
| Hiligsmann (2014) |  |  | ˟ | ˟ |  |  |
| Honkanen (2006) |  |  | ˟ | ˟ | ˟ | ˟ |
| Johansson (2008) |  |  | ˟ | ˟ |  |  |
| Mori (2017) |  |  | ˟ | ˟ | ˟ |  |
| Moriarty (2019) |  |  | ˟ | ˟ | ˟ | ˟ |
| Nshimyumukiza (2013) | ˟ |  | ˟ |  | ˟ |  |
| OMAS (2008) |  |  | ˟ | ˟ | ˟ | ˟ |
| Pega (2016) |  | ˟ | ˟ | ˟ |  | ˟ |
| RCN (2005) |  |  |  | ˟ |  |  |
| Wilson (2017) |  | ˟ | ˟ | ˟ |  | ˟ |
| Zarca (2014) |  |  | ˟ | ˟ |  |  |
| **Abbreviation:** LTC: long-term care; OMAS: Ontario Medical Advisory Secretariat; RCN: Royal College of Nursing  ^1^ See Table 1 in main manuscript for study references. | | | | | | |

The two columns under ‘Population’ record whether the model incorporated migration and entry processes that affect the target population size over time. The two columns under ‘Mortality’ report whether the models incorporated mortality attributable to falls (via immediate fatality or as excess mortality risk) and/or non-fall-related mortality. The two columns under ‘LTC admission’ report whether the models incorporated LTC admission attributable to falls and/or non-fall-related admission. Only those that directly incorporated LTC admission as a separate model state (rather than just include the cost of LTC admission) were marked.

# Implementation level definitions

| **Table S3** Implementation levels by demand and supply dimensions | | |
| --- | --- | --- |
| **Process** | **Demand dimension** | **Supply dimension** |
| *Access* | Uptake (1) | Adoption (2) |
| *Compliance* | Adherence (1) | Fidelity (3) |
| *Sustainability^1^* | Persistence (4) | Maintenance (2) |
| ^1^ The extent to which access and compliance are preserved over time after the initial receipt for interventions requiring ongoing receipt (e.g., exercise). | | |

# References

1. Nyman SR, Ballinger C. A review to explore how allied health professionals can improve uptake of and adherence to falls prevention interventions. British Journal of Occupational Therapy. 2008;71(4):141-5.

2. Li F, Harmer P, Fitzgerald K. Implementing an Evidence-Based Fall Prevention Intervention in Community Senior Centers. Am J Public Health. 2016;106(11):2026-31.

3. Li F, Harmer P, Stock R, Fitzgerald K, Stevens J, Gladieux M, et al. Implementing an evidence‐based fall prevention program in an outpatient clinical setting. Journal of the American Geriatrics Society. 2013;61(12):2142-9.

4. Karlsson L, Lundkvist J, Psachoulia E, Intorcia M, Ström O. Persistence with denosumab and persistence with oral bisphosphonates for the treatment of postmenopausal osteoporosis: a retrospective, observational study, and a meta-analysis. Osteoporosis International. 2015;26(10):2401-11.
